# Using decision trees to characterize verbal communication during change and stuck episodes in the therapeutic process

**DOI:** 10.3389/fpsyg.2015.00379

**Published:** 2015-04-09

**Authors:** Víctor H. Masías, Mariane Krause, Nelson Valdés, J. C. Pérez, Sigifredo Laengle

**Affiliations:** ^1^Department of Management Control and Information Systems, Universidad de ChileSantiago, Chile; ^2^Faculty of Economics and Business, Universidad Diego PortalesSantiago, Chile; ^3^Psychology School, Pontificia Universidad Católica de ChileSantiago, Chile; ^4^Faculty of Psychology, Universidad del DesarrolloSantiago, Chile

**Keywords:** decision trees, significant event, coding system, counseling, pilot teaching method

## Abstract

Methods are needed for creating models to characterize verbal communication between therapists and their patients that are suitable for teaching purposes without losing analytical potential. A technique meeting these twin requirements is proposed that uses decision trees to identify both change and stuck episodes in therapist-patient communication. Three decision tree algorithms (C4.5, NBTree, and REPTree) are applied to the problem of characterizing verbal responses into change and stuck episodes in the therapeutic process. The data for the problem is derived from a corpus of 8 successful individual therapy sessions with 1760 speaking turns in a psychodynamic context. The decision tree model that performed best was generated by the C4.5 algorithm. It delivered 15 rules characterizing the verbal communication in the two types of episodes. Decision trees are a promising technique for analyzing verbal communication during significant therapy events and have much potential for use in teaching practice on changes in therapeutic communication. The development of pedagogical methods using decision trees can support the transmission of academic knowledge to therapeutic practice.

## 1. Introduction

The gap that has long existed between clinical research and clinical practice in psychotherapy has been widely documented (Barlow, 1981; Elliott, 1983a; Talley et al., 1994; Goldfried and Wolfe, 1996; Monger, 1998; Kazdin, 2001; Jiménez, 2002; Krause, 2011). One of the problems currently face by researchers in therapeutic communication is finding analytic techniques that have pedagogical potential for teaching clinical knowledge not only to therapy educators but also to practicing therapists and the patients themselves. Academic research has found that therapy processes include Change Episodes (CE) as well as Stuck Episodes (SE), both of which are significant events (Elliott, 1983b; Mahrer and Nadler, 1986; Elliott et al., 1985; Gonçalves et al., 2009). Whereas CE generate the transformation of the client's subjective perspective regarding him- or herself, her problems and symptoms, SE can be characterized by the temporary detention of the client's change process (Krause et al., 2006; Fernández et al., 2012). However, no work has been published on the use of alternative analytical techniques for transmitting this knowledge, which has been documented in various qualitative and quantitative studies (Brehm and Brehm, 1981; Rice and Greenberg, 1984; Etchegoyen, 1987; Bastine et al., 1989; Grafanaki and McLeod, 1999; Safran and Muran, 2000; Arkowitz, 2002; Timulak and Elliott, 2003; Billow, 2006, 2007; Krause et al., 2006; Miron and Brehm, 2006; Ramírez et al., 2006; Gonçalves et al., 2009; Herrera Salinas et al., 2009; Valdés et al., 2012; Fernández et al., 2012).

Existing research on verbal communication shows that uncovering the communication rules in a therapy process is a multi-dimensional analytical problem. On the one hand, to explore the communication between therapist and patient there are numerous Coding Systems (CS) (Friedlander, 1982; Evans et al., 1984; Cobb and Lieberman, 1987; Lieberman and Cobb, 1987; Mahrer et al., 1988; Stiles, 1992; Wiser and Goldfried, 1996; Connolly et al., 1998; Shaikh et al., 2001; Sirigatti, 2004; Trijsburg et al., 2004; Roussos et al., 2006; Del Piccolo et al., 2011; Rimondini, 2011), which are frameworks that contain a set of variables describing the modes of verbal response occurring in a therapeutic process (Valdés et al., 2010b; Froján Parga et al., 2011). And on the other hand, there are a variety of statistical techniques for analyzing these systems (Mazzi, 2011; Gelo et al., 2013; Mörtl and Gelo, 2015). Finally, there exists a range of theories on interpersonal communication in healthcare settings (Street et al., 2009; Bylund et al., 2012; Wouda and van de Wiel, 2013) and an array of techniques for teaching communication (Berkhof et al., 2011; Bylund et al., 2012).

Studies of CE and SE have used logistic regression to analyze the data gathered during these episodes. Though clinical studies have validated this method of approximation (Harre et al., 1988; Steyerberg et al., 2001), reports have shown that even experienced researchers do not always have the training to properly interpret the results of logistic regression analysis (King et al., 2000; Mood, 2010) or the necessary skills to communicate them (Wouda and van de Wiel, 2012). Thus, there is a lack of alternative pedagogical techniques for transmitting knowledge of verbal communication in therapeutic processes to professionals or students who have relatively little background in statistical modeling. This in turn points up the need for techniques that can create models suitable for teaching purposes that are easy to interpret without losing their analytical potential.

This article proposes the use of decision trees (DT), also called classification and regression trees, to analyze and interpret the communication rules that characterize CE and SE. A DT is “a way to represent rules underlying data with hierarchical, sequential structures that recursively partition the data” (Murthy, 1998, p. 345). It is a technique that learns to recognize patterns in data and has performed well in various areas of application (Laengle, 1992; Quinlan, 1993; Rokach, 2007; Wu et al., 2008). Furthermore, DT's are used as pedagogical support tools to produce easy-to-interpret models generally (Breiman, 2001b; Jormanainen and Sutinen, 2012; Anaya et al., 2013). However, to our knowledge it has not been employed to characterize the verbal communication that takes place during change or SE in therapeutic processes. Some of the advantages that have prompted us to apply DT's to the study of this phenomenon are the following (Zhao and Zhang, 2008, p. 1956):

- They are easy to understand.
- They are easily converted to a set of production rules.
- They can classify both categorical and numerical data (but the output attribute must be categorical).
- There are no a priori assumptions about the nature of the data.

In our exploration of the performance of DT's we apply three different DT generation techniques to the problem of classifying episodes as either CE or SE. Our data source is a linguistic corpus of 8 therapies delivered in individual mode that were coded using the Therapeutic Activity Coding System (TACS) (Valdés et al., 2010b) and CE and SE indicators (Krause et al., 2007). An experiment is conducted to test the three DT models, and the best one forms the basis for a proposed pilot teaching method. This method consists of a series of steps to be used by instructors tasked with introducing students to the recognition of change and SE.

The remainder of the paper is organized as follows. Section 2 provides some basic information on change and SE in the therapeutic process; Section 3 details the experimental method employed; Section 4 sets out the results of the experiments, compares the performance of the different DT tested and presents the 6 steps making up our proposed pilot teaching method; and finally, Section 5 presents our conclusions and discusses some practical implications.

## 2. Change episodes and stuck episodes in the therapeutic process

Much research has been done on the processes of therapeutic communication since the days when Freud, practiced his treatment through words based on techniques learned from Breuer (Breuer and Freud, 1895). Over the last 25 years, research into change processes has been directed at *significant events* or episodes during therapy (Elliott, 1983b; Elliott et al., 1985; Mahrer and Nadler, 1986; Gonçalves et al., 2009), focusing on episodes related to change (Rice and Greenberg, 1984; Bastine et al., 1989; Timulak, 2010; Timulak et al., 2010; Marto, 2012) as well as difficulties during these processes (Ramírez et al., 2006; Herrera Salinas et al., 2009). Events linked with change have received several names such as empowerment events (Timulak and Elliott, 2003), innovative moments (Gonçalves et al., 2009), insight (Rice and Greenberg, 1984), helpful events (Grafanaki and McLeod, 1999), and CE (Bastine et al., 1989; Herrera Salinas et al., 2009; Sánchez, 2012). Also, though less frequently, events related to difficulties in the therapeutic process have been conceptualized as ruptures (Safran and Muran, 2000), refusal (Billow, 2006, 2007), reactance (Brehm and Brehm, 1981; Miron and Brehm, 2006), resistance to change (Arkowitz, 2002), impasse (Etchegoyen, 1987), hindering events (Grafanaki and McLeod, 1999), and SE (Ramírez et al., 2006; Herrera Salinas et al., 2009; Sánchez, 2012), to name a few.

**Figure 1:**
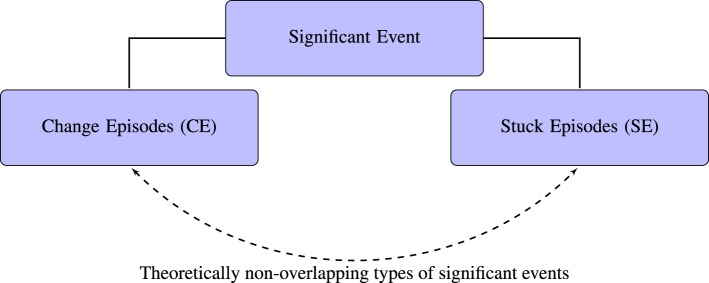
**Change Episodes (CE) and Stuck Episodes (SE) are types of significant events that occur during the process of therapeutic change**.

A review of the theoretical and empirical literature confirms that CE and SE are the two types of significant episodes existing in a therapeutic process (see Figure 1). As noted earlier, during a CE a transformation of the client's subjective perspective regarding him—or herself, her problems and symptoms, and the association of these with the environment takes place (Krause et al., 2007). This involves the development of new forms of interpretation and representation. An SE, by contrast, can be seen as the opposite of a CE, being characterized by the temporary detention of the client's change process due to the persistence in ways of understanding, behavior and emotions related to his or her problem. In both types of episodes the focus of the observation is primarily on the patient, regardless of the actions or omissions of the therapist and their possible strengths or weaknesses. While in CE new meanings are constructed, SE are characterized by the lack of construction of new modes of interpretation or representation. From a general perspective, CE and SE are two different moments in which therapist and patient configure their alliance and their therapeutic relationship (Safran et al., 1990; Valdés et al., 2010a; McCarthy et al., 2011).

The two episode types are both about 3 min long and are determined through trained clinical observation. The analytical problem thus consists in identifying what verbal communication attributes characterize these episode types but the pedagogical problem is how to teach a therapist in training to detect them. This is especially challenging because identifying these moments requires long therapeutic experience or the expert knowledge of a psychotherapist, neither of which a student therapist will have. In what follows we describe an experimental setup for identifying the elements of CE and SE using DT.

## 3. Materials and methods

To build a model capable of classifying CE and SE we designed the experimental setup (depicted in Supplementary Figure 1), which combines qualitative and quantitative data analysis techniques. The method consists of six phases: process recording, data coding, running experiments, calculating performance measures, evaluation of DT via statistical hypothesis testing, and introducing the best of the DT models into teaching and learning. These phases are described in turn below.

### 3.1. Process recording

The analysis unit is the “speaking turns” taken by the therapist and the patient participating in the psychodynamic individual-mode therapy processes conducted for the study. After being told of the scope and objectives of our work, both participants signed consent forms permitting the therapy sessions to be recorded, analyzed and quoted from for research purposes as long as anonymity and confidentiality were maintained. The sessions were held in a room with a one-way mirror to facilitate recording in video as well as audio with 8 trained clinical data analysts acting as observers. The dialogues that took place between the therapist and the patient were transcribed verbatim. The research was approved by the Ethics Committee of the Psychology School belonging to the Pontificia Universidad Católica de Chile and by the Ethics Committee of the Chilean National Fund for Research and Technology (FONDECYT).

### 3.2. Data and coding

The coded database is an aggregate linguistic corpus of the 8 successful therapy processes. Of the 1760 speaking turns registered during these processes, 1003 belonged to 23 SE and 757 belonged to 22 CE. The codification of the independent variables (communicative actions) and the dependent variable (type of episode) is described below.

#### 3.2.1. Codification of independent variables:

The speaking turns were codified in 31 binary categorical variables representing 31 different communicative actions defined by the TACS system (Valdés et al., 2010b) (see Supplementary Table 1). The 8 clinical data analysts observing the sessions determined the presence or absence of these actions. The presence of an action during a speaking turn was codified as a 1 and the absence of an action as a 0.

#### 3.2.2. Codification of dependent variables:

To delimit and codify the CE and SE, the speaking turn in which a CE or SE began had first to be identified. To do this the data analysts used a set of 19 generic CE indicators and 11 qualitative thematic SE indicators (Krause et al., 2007) (see Supplementary Tables 2, 3). A binary nominal variable was defined and assigned a value of 1 for turns occurring during a CE and 0 for turns occurring during an SE.

### 3.3. Running experiments

As noted above, three different DT were used to analyze the communicative actions for classifying episodes as either CE or SE. Our first choice was the C4.5 algorithm because the trees it creates are easy to interpret and perform well, but for purposes of comparison we also used the NBTree and REPTree algorithms (see Supplementary Table 4). To estimate the performance of the DT's, we applied the stratified 10-fold cross-validation approach (Japkowicz and Shah, 2011; Purushotham and Tripathy, 2012), in which “each fold is stratified so that they contain approximately the same proportion of class labels as the original dataset” (Purushotham and Tripathy, 2012, p. 684).

### 3.4. Performance measures

The results of the DT models were classified by a confusion matrix (Rokach, 2007) (see Supplementary Figure 2). Based on this matrix, five DT performance measures denoted Overall Accuracy, Precision, Recall, Matthew's Correlation Coefficient (MCC) (Matthews, 1975), and Area Under the Receiver Operating Characteristic curve (ROC Area) were defined by formulas (see Supplementary Table 5). Thus, performance was measured by comparing the values obtained for these indicators.

### 3.5. Statistical evaluation of dt's performance

Two tests were used to evaluate the performance of DT's: the Cochran's *Q*-Test (Sheskin, 1997) and the McNemar's Test (Bostanci and Bostanci, 2013). The null hypothesis (*H*_0_) of the first test was that they performed similarly whereas the alternative hypothesis (*H*_1_) was that they did not, that is, that they performed differently. If the null hypothesis of Cochran's *Q*-Test is rejected (i.e., the DT's have different performance), then we applied the second test for each pair of models generated by the algorithms C4.5, NBTree, and REPTree. Thus, the null hypothesis (*H*_0_) of the McNemar's Test was used to determinate if two DT's have a similar performance, whereas the alternative hypothesis (*H*_1_) was that they did not.

### 3.6. Teaching and learning the best dt model

Finally, we propose the 6 steps of our pilot teaching method for use by therapy instructors introducing students to the DT model that performed best. These steps were devised by the authors of the present article based on group brainstorming, a qualitative technique for generating ideas that has been used in educational and health professional contexts (Burnard, 1988; Handfield-Jones et al., 1993; Isaksen, 1998; Byron, 2012).

## 4. Results

This section sets out the performance measure results for the three DT's (Section 4.1), displays the tree generated by the best-performing algorithm and offers presents the steps in the pilot teaching method (both in Section 4.2).

### 4.1. Comparison and evaluation of dt's performance

The performance measure results for the three DT's on the problem of classifying CE and SE speaking turns are summarized in Table 1. As can be seen, the C4.5 algorithm achieved the highest values for the Precision, Recall, ROC Area, and MCC measures. In addition, the DT it generated was smaller and had fewer leaves than the trees produced by the other two algorithms. Note furthermore that although the DT generated by NBTree performed better than the one created by REPTree, it was also much larger.

**Table 1:** **The Performance of DT's in classifying significant events can be grouped in size of tree (tree size and number of leaves) and prediction capabilities**.

**Comparison Criteria**	**Measure**		**C4.5**	**NBTree**	**REPTree**
DT's characteristics	Tree size		29	151	39
	Number of leaves		15	76	20
Performance measures	Correctly classified instances		1,166	1,123	1,095
			66.25%	63.80%	62.21%
	Incorrectly classified instances		594	637	665
			33.75%	36.20%	37.79%
	Precision	(AVG)	0.71	0.65	0.61
	Recall	(AVG)	0.66	0.63	0.62
	ROC Area	(AVG)	0.66	0.60	0.63
	MCC	(AVG)	0.32	0.24	0.20

To determine whether differences in performance between the algorithms were statistically significant, we conducted the Cochran's *Q*-Test. The null hypothesis (*H*_0_) was rejected (*Q* = 24.43 with *p* = 0.000), meaning that performances of DT's were statistically different. To determine whether the differences in performance between each pair of algorithms were statistically significant, we conducted the McNemar's Test. The null hypothesis (*H*_0_) was that there were no statistically significant performance differences between two DT's. The null hypothesis was rejected between C4.5 and NBTree (*T* = 16.32 and *p* = 0.000), and between C4.5 and REPTree (*T* = 18.46 and *p* = 0.000). Additionally, was accepted the null hypothesis between NBTree and REPTree (*T* = 3.23 and *p* = 0.07). These results show that the performance of C4.5 was indeed statistically different and superior, while the performance levels of REPTree and NBtree were similar and inferior. Furthermore, the tree generated by C4.5 was smaller than the other two as well as performing better makes it particularly suitable for a pedagogical setting.

### 4.2. Teaching and learning using dt model

As just suggested above, for teaching purposes it is not only a DT's performance that matters but also its size and the number of rules it requires. This is so because smaller size and fewer leaves mean that the graph a student will have to learn to interpret will have fewer rules and objects. The smaller size and fewer leaves of the C4.5 DT will thus aid in simplifying the interpretability of the model obtained.

**Figure 2:**
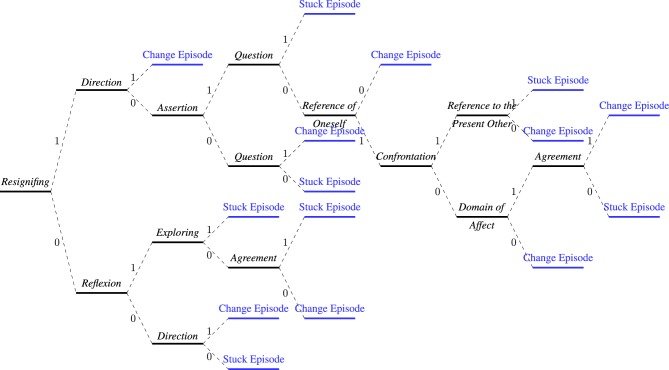
**Visualization of decision tree**. The black thick line (split nodes) represent communicative actions (independent variables), the dashed lines indicate the variable values (communicative action present equal to 1, communicative action absent equal to 0), and the blue thick line (leaf nodes) indicate the type of episode, that is, CE or SE (dependent variable). Each path from the root node to the leaves is a communicative rule that classifies speaking turns as CE or SE. Note finally that some formal aspects of the DT have been omitted here in order to focus on the decision rules acquired with the model (exemplified in Supplementary Table 8); further information on the model's statistical properties can be found in Podgorelec et al. (2002), Lee et al. (2009), Perner (2011), and Kotsiantis (2013).

The DT graph generated by C4.5 is displayed in Figure 2. On the basis of this graph and the group brainstorming process, we drafted the 6 steps in our pilot teaching method for psychotherapy instructors introducing clinical therapy students to this DT. In particular, the brainstorming aimed at generating a method for teaching those with minimal knowledge of statistics to identify EC and SC. The steps themselves are presented in Supplementary Tables 6, 7, 8.

## 5. Discussion

The purpose of this study was to use the DT technique for analyzing and generating easily interpretable models of verbal communication during significant moments in therapeutic processes. These processes involve changes in subjective patterns of interpretation and development of new comprehensive theories of oneself (Winkler et al., 1989; 1993; Dagnino et al., 2012), but the construction of such change processes occurs at the level of the conversation between patient and therapist.

Tests were conducted of different DT's before settling on a model that was able to correctly classify 66% of speaking turns as belonging either to a CE or an SE. However, we believe that further research, perhaps using a different CS (see Friedlander, 1982; Evans et al., 1984; Cobb and Lieberman, 1987; Lieberman and Cobb, 1987; Mahrer et al., 1988; Stiles, 1992; Wiser and Goldfried, 1996; Connolly et al., 1998; Shaikh et al., 2001; Sirigatti, 2004; Trijsburg et al., 2004; Roussos et al., 2006; Del Piccolo et al., 2011; Rimondini, 2011), would improve its performance and therefore also that of the model used with the pilot teaching method that was proposed. The idea was to link the development of better classification models to better models of teaching.

One of the interesting findings of our DT model is that Resignifying is the most important variable for classifying a speaking turn as a CE or an SE. In logistic regression analysis there is little consensus and various criteria for evaluating the importance of a predictor (Thomas et al., 2008). By contrast, the DT quickly shows clinical researchers and student therapists which variable is most important. Although it has been previously reported that the Resignifying communicative action is the most frequently occurring variable in the final phase of therapy (Dagnino et al., 2012), the present study is the first to show that the variable is necessary but not sufficient for classifying CE and SE. Also, the production rules show that Resignifying in the presence of other communicative actions may be characteristic of an SE. This may seem contradictory if we assume that the presence of Resignifying by itself is associated with a CE. Using DT thus shows that the presence of Resignifying in combination with other communicative actions is what distinguishes CE from SE, not Resignifying alone.

The above result indicates how a DT reveals the complexity of the combination of attributes in a therapist-patient conversation. This is consistent with theoretical concepts of psychotherapy according to which the purpose of therapy is to generate new meanings through therapeutic conversation (Watzlawick et al., 1974; Watzlawick, 1976; de Shazer, 1979; Capps, 1990; Pesut, 1991), with resignifing as one of various attributes that contribute to changes. In future research we intend to carry out a pilot study of the application of the proposed teaching method to the measurement of student learning using DT's. Only this way can the potential of DT's for introducing students to the complexity of verbal communication in therapy be empirically tested.

### 5.1. Practice implications

Training in communication skills is an educational value (Rotthoff et al., 2011) that is into practice with the available technology. Based on results using data mining, we posit that DT techniques can be introduced into both clinical research on therapeutic communication and the practice of counseling or therapy. By using different scales of measurement as independent variables, DT's are able to analyze data obtained using different existing CS (Evans et al., 1984; Mahrer et al., 1988; Wiser and Goldfried, 1996; Connolly et al., 1998; Shaikh et al., 2001; Sirigatti, 2004; Trijsburg et al., 2004; Roussos et al., 2006), and through its ability to generate a graph of the induced model and production rules, DT's also have potential for use by various actors in addition to researchers who explore issues of communication in therapy. In practical terms, DT techniques are a valuable new pedagogical tool for the study and teaching of verbal communication in therapeutic processes.

### 5.2. Research limitations

The method used in this paper has two principal limitations. First, as some studies have demonstrated, DT can be unstable (Last et al., 2002; Kitsantas et al., 2007) with the consequence that labeling and adding new examples to the training set may generate changes in the tree originally obtained. To improve the results presented here a stability analysis of the tree could be undertaken similar to the one conducted in Dwyer and Holte (2007). This would provide information on the tree's stability in addition to the evaluation of its performance already done here.

The second limitation stems from the fact that there exists a variety of measures for analyzing variable importance (Rokach and Maimon, 2005). For example, the C4.5 algorithm used here applies an approximation heuristic to determine which predictor variable is the most important on the basis of the highest gain ratio. Researchers contemplating extensions of this work could opt for other criteria such as the gain rate or the Gini index (Raileanu and Stoffel, 2004). Another alternative would be to utilize DT algorithms such as Random Forest (Breiman, 2001a) that incorporate a more robust variable importance measure, but this may involve important tradeoffs. In the Random Forest case, the final output is constructed with 500 trees and is thus not humanly readable. In effect, the algorithm is a *black box* in which what is gained in robustness is lost in interpretability.

### 5.3. Conclusion

Based on the exploratory and comparative results of our study, we conclude that DT techniques have great potential for classifying the modes of verbal responses in therapeutic communication into CE or SE. Greater accuracy may be obtainable through further research into the performance of other CS in classification or other problems. In either case, the techniques considered must take into account the needs of teaching and practical learning. The ultimate goal is to find methods that recognize and reinforce the fundamental concept that therapy using treatment through words is based on a unified paradigm of teaching, learning and research.

## Author contributions

VM designed the research, VM and SL conducted the empirical tests. MK, NV, and JCP carried out the data collection and studied the research domain. VM, MK, NV, and JCP, and SL wrote the paper. All authors read and approved the final manuscript.

### Conflict of interest statement

The authors declare that the research was conducted in the absence of any commercial or financial relationships that could be construed as a potential conflict of interest.
