# Supplementary material for: Using decision trees to characterize verbal communication during change and stuck episodes in the therapeutic process
Source: Front Psychol. 2015 Apr 9;6:379. doi: 10.3389/fpsyg.2015.00379 (PMC4391223; doi:10.3389/fpsyg.2015.00379)
Supplement: Supplementary file 1 [file Table1.PDF]

Frontiers in Psychology

Method Article
14 January 2015

Supplementary Material: Using Decision Trees to Characterize Verbal Communication During Change and Stuck Episodes in the Therapeutic Process

Correspondence*:
,

1 SUPPLEMENTARY TABLES AND FIGURES

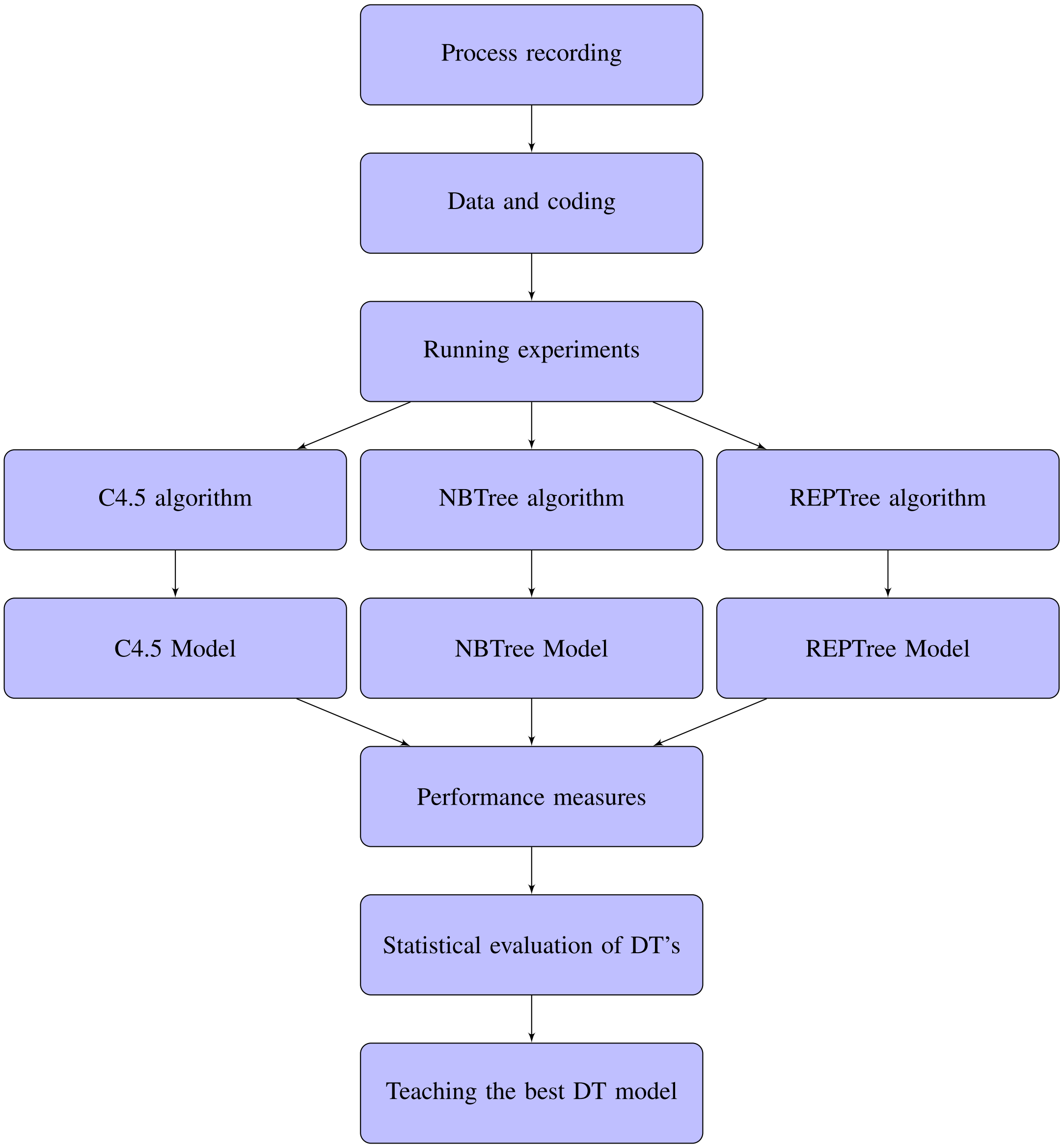

Supplementary Figure 1. Experimental setup consists of six phases: it starts with the process recording of 8 trained clinical data and ends with teaching the best Decision Tree (DT) model, which was statistically selected.

Supplementary Table 1. The Therapeutic Activity Coding System (TACS) has 5 dimensions, each one associated with several communicative actions of which there are 31 in all.

Dimension	Meaning	Communicative Action
**Basic Form**	This dimension of analysis refers to the formal structure of the verbalization, differentiating among:	1) Agreement, 2) Assertion, 3) Denial, 4) Question, and 5) Direction.
**Intention**	This dimension of analysis refers to the communicative purpose expressed by the speaker's words.	1) Exploring, 2) Attuning, and 3) Resignifying.
**Technique**	Methodological tools evident in the communicative action. Some of these techniques coincide with classic psychotherapeutic techniques and others are part of everyday communication.	1) Justification, 2) Self–disclosure, 3) Confrontation, 4) Advice, 5) Interpretation, 6) Imagery, 7) Information, 8) Narration, 9) Labeling, 10) Paradox, 11) Reflection, 12) Reinforcement, 13) Summary, and 14) Roleplaying.
**Domain**	Whether the focus of the therapeutic work is oriented to:	1) the world of ideas (cognitive), 2) the world of action (behavior), and 3) the world of affect (emotion).
**Reference**	Whether the verbalization is aimed at:	1) Oneself (the patient or the therapist); 2) someone else present in the session (the patient, the therapist or some other person), 3) a third party outside the session, 4) the therapeutic relationship, 5) the relationship with a third party outside of the session, and 6) to a neutral subject.

Supplementary Table 2. Qualitative indicators of Change Episodes (CE). This type of content qualifies the therapeutic ongoing change related to the client's transformations in his/her subjective perspective regarding problems, symptoms, and their relationship with the environment in which they occur. The change indicators evolve throughout the therapeutic process, showing a sequential nature and a progressive movement toward higher–ordered levels of construction of new meanings (1).

Level of change	Qualitative indicators of Change Episodes (CE)
**Level 1: Initial consolidation of the structure of the therapeutic relationship**	• Acceptance of the existence of a problem<br>• Acceptance of his/her limits and of the need for help<br>• Acceptance of the therapist as a competent professional<br>• Expression of hope<br>• Questioning of habitual understanding<br>• Expression of the need for change<br>• Recognition of his/her own participation in the problems
**Level 2: Increase in permeability towards new understandings**	• Discovery of new aspects of self<br>• Manifestation of new behavior or emotions<br>• Appearance of feelings of competence<br>• Establishment of new connections<br>• Reconceptualization of problems and/or symptoms<br>• Transformation of valorizations and emotions in relation to self or others
**Level 3: Construction and consolidation of a new understanding**	• Creation of subjective constructs of self through the interconnection of personal aspects and aspects of the surroundings, including problems and symptoms<br>• Founding of the subjective constructs in own biography<br>• Autonomous comprehension and use of the context of psychological meaning<br>• Acknowledgement of help received<br>• Decreased asymmetry between patient and therapist<br>• Construction of a biographically grounded subjective theory theory of self and of his/her relationship with surroundings

Supplementary Table 3. Qualitative indicators of Stuck Episodes (SE). This type of content qualifies the argumentative issues that persist in the patient's discourse and do not contribute to the focus on change. Stuck episodes are characterized by temporary stalling of the patient's process of change due to the repetition of a dysfunctional pattern during the therapeutic process (2).

**Qualitative indicators of Stuck Episodes (SE)**
• Denies or minimizes the existence of a problem<br>• Denies need of help and does not accept own limitations<br>• Expresses hopelessness (demoralisation)<br>• Avoids responsibility for own acts<br>• Expresses feelings of incompetence<br>• Expresses an increase in fear or ambivalence related to the impending change<br>• Attributes own problems to others<br>• Resists thinking about new possibilities for (behavior, thoughts or emotions)<br>• Questions the professional competence of the therapist<br>• Resists establishing a relationship between symptoms, emotions, and behavior<br>• Resists changing concepts related to the problem or symptom

Supplementary Table 4. Decision tree induction algorithms used in experiments.

DT Algorithm	Description	Source
**C4.5**	Uses information entropy to create a classification problem tree. The algorithm can generate trees that may or may not be pruned	(3)
**NBTree**	Creates a tree whose leaves are constructed using naive Bayes classifiers	(4)
**REPTree**	Reduced–Error Pruning is a decision tree learner using CART's minimal cost complexity pruning	(5)

		Prediction outcome		
		positive	negative	
Actual value	positive	TP	FN	$TP + FN$
	negative	FP	TN	$FP + TN$
		$TP + FP$	$FN + TN$	

Supplementary Figure 2. Confusion matrix for classifying the predictions each model. TP : is the number of correct predictions that an instance is positive (*true positive*), FN : is the number of incorrect predictions that an instance is negative (*false negative*), FP : is the number of incorrect predictions that an instance is positive (*false positive*), and TN : is the number of correct predictions that an instance is negative (*true negative*).

Supplementary Table 5. Performance measures in binary classification. Formulas are based on the confusion matrix classifications of Supplementary Figure 2. Each measure varies between 0 and 1, excepting measure MCC which takes values between -1 and $+1$. In every case, values close to +1 indicate a good performance.

Measure	Formula
**Accuracy**	$\frac{TP + TN}{TP + FP + FN + TN}$
**Precision**	$\frac{TP}{TP + FP}$
**Recall**	$\frac{TP}{TP + FN}$
**ROC Area**	$\frac{1}{2}\left(\frac{TP}{TP + FN} + \frac{TN}{TN + FP}\right)$
**MCC**	$\frac{TP \cdot TN - FP \cdot FN}{\sqrt{(TP + FP)(TP + FN)(FP + TN)(FN + TN)}}$

Supplementary Table 6. Pilot teaching method steps. All of these tasks can be performed individually or in groups. Although we have not yet done a quantitative evaluation of the pilot method produced by the group brainstorming process, in qualitative terms it has so far been well received by both academics and students. In future research we hope to measure the learning curve for the DT model based on the results using the pilot teaching method.

Teaching Step (TS)	Objetive (O)	Question (Q)	Answer (A)
**TS 1:**	Introduce the visual structure of a Decision Tree (DT) graph (10 minutes).	**Q:** What is the general structure of a DT graph?	**A:** A DT is a graph without loops consisting of a root node, internal (split) nodes and terminal (leaf) nodes connected by valued edges. It is organized hierarchically with the root node located at the left of the graph. Arcs extending from the root node connect to two internal (split) nodes each of which can connect to two other internal (split) nodes or two terminal (leaf) nodes. Whereas the root and internal (split) nodes represent independent variables, the terminal (leaf) nodes represent a class (i.e., the answer). Finally, each arc or value link represents the value taken by the variable to realize the class prediction. Visually, the DT should be described as having the appearance of an tree with a trunk, branches and leaves.
**TS 2:**	Define the meaning of the nodes, leaves and value links in the domain of application (10 minutes).	**Q:** In concrete terms, what do the nodes and links represent in the context of verbal communication?	**A:** The nodes represent a communicative action (see Supplementary Table 7) that characterizes CE and SE occurring during therapy sessions. Communicative actions may be present or absent in a speaking turn that is part of a CE or SE. The links represent the presence or absence of a given communicative action in a given speaking turn during a CE or SE. The various possible communicative actions are described in Figure 2.
**TS 3:**	Help identify which is the most important communicative action (variable) for classifying episodes as CE or SE (classes) (5 minutes).	**Q:** Which is the most important communicative action for characterizing CE and SE?	**A:** Show the DT model and point out that the root node of the tree, representing the action of *Resignifing*, is the most important communicative action and the key one for classifying episodes as CE or SE.

Continued on next page

Table 6 – continued from previous page

Teaching Step	Objetive	Question	Answer
**TS 4:**	Help extract the communication rules from the DT.	**Q:** What verbal communication rules characterize the CE and SE?	**A:** Each path from a root node to a leaf node represents a production rule that characterizes the episode types. As can be seen, the DT has 15 leaves and therefore 15 of these paths passing through internal nodes. Each such path consists of a conjunction of communicative actions (variables) whose presence or absence characterizes a speaking turn belonging to a CE or SE. The conjunction of variables and values in a production rule is called a "Rule Antecedent" (the "IF" part) and the classes to be predicted (leaf nodes) are called "Rule Consequent" (the "THEN" part) (see Supplementary Table 8)
**TS 5:**	Exemplify the rules extracted from the tree with concrete examples taken from therapy (30 to 45 minutes).	**Q:** How can the rules of verbal communication during CE and SE be exemplified?	**A:** Each individual decision rule can be exemplified using the verbatim transcriptions of the therapeutic communication between therapist and patient. This can be done by identifying speaking turns (instances) correctly classified by rules and use them as examples. One approach would be to present 1 to 4 examples per rule so that the student associates the rules with empirical material. An example of a table containing therapy rules and their examples is given in see Supplementary Table 8.
**TS 6:**	Assignments: Evaluate how well the students learned the DT (45 minutes for each evaluation).	**Q:** How can student learning be evaluated?	**A:** Six assignments for evaluating student learning have been defined, consisting of the following tasks: a) Draw the DT that classifies episodes as CE or SE, b) Define what the nodes and links represent in the domain of application, c) Identify which node is the most important one for classifying episodes as CE or SE, d) Extract and state the communication rules of the DT, e) Classify 15 CE or SE from the verbatim text, and, f) Justify the classification using a rule extracted from the DT.

Supplementary Table 7. Definition of communicative actions characterizing a Change Episodes (CE) or Stuck Episodes (SE) discovered by C4.5 DT model.

Communicative action	Definition
**Agreement**	Recognizes the truth of another's statement (e.g., "Right," "Yes," "Of course," "That could be," "mhm").
**Assertion**	Expresses something that is considered to be true (e.g., "but he/she still misses her, it could be different," "not making any promises," "impossible").
**Confrontation**	Methodological resource used to confront the other or oneself with certain assertions (e.g., "it is very difficult to keep up the same lifestyle as before and do the same things you used to do"; "whether it is difficult or not, that is just the way it is, period.")
**Direction**	Encourages the other towards cognitive, emotional or behavioral action (e.g., "Think about what I just told you," "Hold on to that feeling," "Look at your husband when you are talking to him").
**Domain of Affect**	In this Domain, the focus of the therapeutic work is primarily on conduct and is of a behavioral nature.
**Exploring**	This communicative action aims to: a) ask for or provide contents that are unknown to the participants; b) to clarify contents; and/or c) to direct attention and efforts towards a specific topic of conversation.
**Question**	A request for specific information (e.g., "And what was he/she complaining about then?" "And how did that feel at that time?").
**Reference to Oneself**	The verbalization refers to oneself and is formulated in the first–person singular or first–person plural as a social group.
**Reference to the Present Other**	The verbalization refers to a present other and is formulated in the second–person singular or plural. This category does not include verbalizations referring to the therapeutic relationship.
**Reflection**	Methodological resource used as a mirror to display the emotional, cognitive and/or behavioral states of the other (e.g., "Also, you look like you are bored and tired").
**Resignifying**	This communicative action aims to: a) generate or b) consolidate new meanings.

Supplementary Table 8. Classification rules and examples of verbal communication during change and stuck episodes (CE and SE) in the therapeutic process. The decision tree model that performed best was generated by the C4.5 algorithm. It delivered 15 rules in a form of IF–THEN rules that are easily readable.

Rule	If	Then	Example (in spanish)	Translated example
**1**	(Resignifing=0) AND (Reflection=0) AND (Direction=1):	CE	*Therapist*: "Entonces, yo lo que le quiero decir es que en esta clínica puede continuar una terapia, pero que nos pongamos de acuerdo que vamos a trabajar en estos aspectos que yo le he dicho, cómo resolver esta situación, cómo es lo que usted piensa, cómo ha sido su pasado con respecto a esto y, que nos demos un tiempo, que yo he pensado en 20 sesiones, en este horario, para pensar sobre esto" (T5S4:223).	*Therapist*: "So what I want to tell you is that you can continue therapy at this clinic, but we will have to agree to work on the aspects I mentioned, that is, how to resolve the situation, what you think, what is your past experience on this. We'll have to define a schedule, I'm thinking in terms of 20 sessions at this same time to think about this" (T5S4:223).
**2**	(Resignifing=0) AND (Reflection=1) AND (Exploring=0) AND (Agreement=0):	CE	*Therapist*: "Se sentía cohibida, inhibida con él, reprimida tal vez" (T2S3:71).	*Therapist*: "She felt uneasy, inhibited when she was with him, repressed maybe" (T2S3:71).
			Therapist: "Ah, tú escuchas que yo te estoy diciendo una falla" (T15S4:319).	*Therapist*: "I see, what you're hearing is that I'm mentioning a fault to you" (T15S4:319).
**3**	(Resignifing=1) AND (Direction=0) AND (Assertion=0) AND (Question=1):	CE	*Therapist*: "O sea, esta idea de los hombres son 'básicos', ¿Viene de la mamá?" (T10S10:352).	*Therapist*: "So, this idea that men are 'basic' beings comes from your mom?" (T10S10:352).
			Therapist: "Pero, ¿le queda claro que es una manera de protegerse y darse seguridad?" (T10S4:701).	*Therapist*: "But, is it clear to you that this is a way to protect yourself and gain security?" (T10S4:701).
**4**	(Resignifing=1) AND (Direction=0) AND (Assertion=1) AND (Question=0) AND (Reference to Oneself=0) AND (Confrontation=0) AND (Domain of Affect=0):	CE	*Therapist*: "Claro, y así como usted, pese a todas las imperfecciones de este espacio, todas las imperfecciones o frustraciones del espacio, ha sido capaz de tener esta conexión, de recibir, de sentirlo como un regalo, ehh entonces eso nos da luces para poder pensar que otras situaciones que usted ve, o que usted le puede encontrar mucho defectos, puede…ehh, ser la realidad así y finalmente buena" (T17S11:588).	*Therapist*: "Right, and because you, despite all the imperfections of this space, all the imperfections or frustrations of this space, have managed to have this connection, to receive, to feel it as a gift, um, so that gives us some clues which suggest that other situations you see, where you can identify lots of defects…um, though reality can be like that, it can eventually be good" (T17S11:588).

Continued on next page

Table 8 – continued from previous page

Rule	If	Then	Example (in spanish)	Translated example
			Therapist: "En realidad me preocupa más…que yo sé que el medicamento no produce muchos efectos colaterales pero, más me preocupaba que la hiciera a usted recordar viejos fantasmas también con los medicamentos, porque usted me ha hablado de varios tipos de agresiones que ha recibido…ahora me está hablando de sus agresiones sexuales, y también me ha hablado de las agresiones medicamentosas que usted siente que ha habido en usted y en su familia" (T1S4:204).	*Therapist*: "Actually, I'm more concerned about…I know the medication doesn't have many side effects, but I was more worried that the medications could make you recall old ghosts, because you've told me about several types of aggressions you've been victim to…now you're telling me about sexual attacks, and you've also mentioned the medication abuse that you feel has been present in your life and in your family" (T1S4: 204).
**5**	(Resignifing=1) AND (Direction=0) AND (Assertion=1) AND (Question=0) AND (Reference to Oneself=0) AND (Confrontation=0) AND (Domain of Affect=1) AND (Agreement=1):	CE	*Patient*: "Sí, me imagino que a lo mejor es así, y creo que con él no fui atrevida, no en el sentido de grosera, como que no fui atrevida con él. Bueno, él me culpaba y en el fondo creo que no soy culpable, lo que pasa es que creo que él no reconoce su situación; él se fue a vivir sólo y de hecho si se fue a vivir sólo era porque quería tener la independencia, la libertad para hacer otras cosas en su vida. O sea, yo creo que eso provocó esto" (T2S3:68).	*Patient*: "Yes, I imagine that may be so, and I think that I wasn't daring enough with him, [it's not that I was insolent], it's like I wasn't daring enough with him. Well, he blamed me and deep down I think it's not my fault, what happens is that I think he doesn't recognize his situation; he went to live on his own and in fact he only did it because he wanted the independence, the freedom to do other things in his life. So, I think that caused this" (T2S3:68).

Continued on next page

Table 8 – continued from previous page

Rule	If	Then	Example (in spanish)	Translated example
			Patient: "Claro, sí, y bueno, ahora mi esposo como que yo pienso que él también se maneja así, como que él me maneja con algo de castigo… o sea ahora que me doy más tiempo para pensar creo que sí porque, por ejemplo, sabe que me afecta que eche mano a los niños, que los trate mal, y cuando él tiene un conflicto conmigo se desquita con los niños, entonces yo eso lo asumo como un castigo; antes por ejemplo, me pegaba, ahora no, después se desquitaba con los niños, o si no por ejemplo, cuando teníamos que discutir y él ve que no tiene fundamento y yo si lo tengo, se para y me deja hablando sola; entonces yo también eso lo tomo como un castigo, porque como que me deja sola ahí, como 'haz lo que quieras sola nadie te va a escuchar'… entonces yo ahora pienso que él me manejaba un poco con eso" (T5S10:95).	*Patient*: "Right, yes, well, I guess my husband does things like that, it's like he controls me with threats of punishment … I mean, now that I'm giving myself more time to think I believe that is the case, because, for example, he knows it affects me when he lays his hands on the children, when he mistreats them, and when he has a conflict with me he takes it out on the children, so I perceive that as a punishment; in the past, for instance, he used to hit me, but not anymore, later on, he would take it out on the children… or, for instance, when we have a quarrel and he sees he has no arguments but I do, he stands up and leaves me speaking alone; so I take that as a punishment too, because he leaves me alone there, as if telling me 'do what you want on your own, nobody will listen'… so now I think that he controlled me a little with this" (T5S10:95).
**6**	(Resignifing=1) AND (Direction=0) AND (Assertion=1) AND (Question=0) AND (Reference to Oneself=0) AND (Confrontation=1) AND (Reference to the Present Other=0):	CE	*Therapist*: "Esa es la parte que Usted no quiere, 'no me hablen de eso' como que me dijera '¿sabe? no me hable de eso, no quiero sentirme vulnerable, no quiero ni por un ratito' " (T18S1:191).	*Therapist*: "That is the part that you don't want, 'don't talk to me about that', it's as if you were telling me 'you know? don't talk to me about that, I don't want to feel vulnerable, not even for a second' " (T18S1:191).
**7**	(Resignifing=1) AND (Direction=0) AND (Assertion=1) AND (Question=0) AND (Reference to Oneself=1):	CE	*Patient*: "Sí, (tono de tristeza) me cuesta, me cuesta y me doy cuenta que sigo girando en redondo, pienso bien, actuó mal, pienso bien, actuó mal, pienso en lo que necesito" (T17S05:269).<br><br>*Patient*: "Ya que cuando llegué aquí, llegué cansado pero como mentalmente, como que estaba todo encerrado" (T15S15:487).	*Patient*: "Yes, (sad tone) it's hard for me, it's hard for me and I realize I'm going round in circles, I think well, I make a mistake, I think well, I make a mistake, I think about what I need" (T17S05:269).<br><br>*Patient*: "When I got here I was tired, but mentally, it's like everything was locked up inside" (T15S15:487).

Continued on next page

Table 8 – continued from previous page

Rule	If		Then	Example (in spanish)	Translated example
**8**	(Resignifing=0)<br>(Reflection=0)<br>(Direction=0):	AND<br>AND	SE	*Patient*: "Pero no funciona como acá, como un equipo; sabe, lo que pasa es que acá, independientemente de lo que pase, independientemente que yo esté como usted decía dos años tomando medicamentos, o me pase la vida, va más allá de eso, no me molesta eso... vi vulnerado mi derecho como paciente, porque a lo mejor como usted dice, usted me dio un diagnóstico, ¿no es cierto?" (T1S5:57).	*Patient*: "But it doesn't work like it does here, like a team; you know, the thing is that here, regardless of what happens, no matter if I've been taking medications for two years, like you said, or if I spend my whole life doing that, it goes beyond that, it doesn't bother me... I felt that my rights as a patient were violated, because, maybe, like you say, you gave me a diagnosis, right?" (T1S5:57).
**9**	(Resignifing=0)<br>(Reflection=1)<br>(Exploring=0)<br>(Agreement=1):	AND<br>AND<br>AND	SE	*Therapist*: "Ya, o sea que para usted puede ser molesto que usted me hable 'de usted' [formal] y yo no le hable nada de mí" (T10S2:265).<br><br>*Therapist*: "Sí, sí, y aquí se está sintiendo incómoda y yo la estoy haciendo sentir incómoda, y a lo mejor, la duda es si lo estoy haciendo a propósito o no, y si este propósito es bueno o no" (T2S9:148).	*Therapist*: "Okay, so for you it can be annoying to address me as 'you' [formal] and that I don't share anything about myself with you" (T10S2:265).<br><br>*Therapist*: "Yes, yes, and at this point you're feeling uncomfortable and I'm making you feel uncomfortable, and maybe, the question is whether I'm doing this on purpose or not, and whether this purpose is good or not" (T2S9: 148).
**10**	(Resignifing=0)<br>(Reflection=1)<br>(Exploring=1):	AND<br>AND	SE	*Therapist*: "Fíjese, que de nuevo vamos a tener que tocar esto de que usted con cierta frecuencia me pregunta '¿me entiende?, ¿me entiende?'... fíjese que a mí me hace pensar que usted tiene dudas si se expresa bien o no" (T10S2:212).	*Therapist*: "Look, we'll have to refer to this thing you do again, you often ask me 'do you understand? do you understand?'... look, this makes me think that you have doubts about whether you express yourself well" (T10S2:212).
**11**	(Resignifing=1)<br>(Direction=0)<br>(Assertion=0)<br>(Question=0):	AND<br>AND<br>AND	SE	*Patient*: "A lo mejor te lo aceptaría antes, ahora no" (T10S2:522).	*Patient*: "Maybe I would have accepted that from you before, but not now" (T10S2:522).

Continued on next page

Table 8 – continued from previous page

Rule	If	Then	Example (in spanish)	Translated example
**12**	(Resignifing=1) AND (Direction=0) AND (Assertion=1) AND (Question=0) AND (Reference to Oneself=0) AND (Confrontation=0) AND (Domain of Affect=1) AND (Agreement=0):	SE	*Therapist*: "Ya, a lo mejor esa cámara y esas personas que están al otro lado del espejo también son molestas, pero pareciera ser que sus enojos a lo mejor los hubiera canalizado hacia el doctor de allá atrás, cosa que yo le agradezco, porque así se enoja con él y no se enoja conmigo de alguna manera; pero yo tengo la impresión que también pudiera acá usted tener susto de que me preocupa más la cámara o las personas que están mirando al otro lado, que su salud o su bienestar" (T1S5:66).	*Therapist*: "Okay, maybe that camera and the people behind the mirror are annoying too, but it would seem you've channeled your anger towards the doctor at the back, which I'm thankful for, because in a way you get mad at him and not at me, but I'm under the impression that here you may be afraid I could be more concerned about the camera or the people behind the mirror than about your health or your well–being" (T1S5:66).
			Therapist: "Usted parece que siente que es tan difícil, se le hace tan difícil que parece que pierde toda la esperanza, que nadie la puede ayudar, que esto tampoco la puede ayudar, a pesar de que lo ha pasado bastante mal este último tiempo, y esta nueva oportunidad de tratamiento, de terapia, tampoco la puede ayudar porque es tan grave lo que tiene, y al contrario, se quedaría [en terapia] si no [insiste en] regresar con las pocas cosas buenas que preserva a como de lugar" (T5S6:96).	*Therapist*: "You seem to feel it's so hard, it's so hard for you that it seems that you lose all hope, that no one can help you, and that this can't help you either, even though you've been having quite a hard time lately, and this new treatment opportunity, this therapy, can't help you either because your condition is too serious, and on the contrary, you would stay [in treatment] if you couldn't come back with the few good things that you preserve at all costs" (T5S6:96).
**13**	(Resignifing=1) AND (Direction=0) AND (Assertion=1) AND (Question=0) AND (Reference to Oneself=0) AND (Confrontation=1) AND (Reference to the Present Other=1):	SE	*Therapist*: "No me diga que no estamos enojados, si usted está evidentemente enojada" (T1S11:203).	*Therapist*: "Don't tell me that we[you] aren't angry when you evidently are" (T1S11:203).
			Therapist: "No es cierto como tú le dices, 'haz lo que tú quieras' " (T15S9:477).	*Therapist*: "It's not true what you say to him, 'do what you want' " (T15S9:477).

Continued on next page

Table 8 – continued from previous page

Rule	If		Then	Example (in spanish)	Translated example
**14**	(Resignifing=1) (Direction=0) (Assertion=1) (Question=1):	AND AND AND	SE	*Therapist*: "¿Sabe? cuando yo le digo estas cosas, a mí me da la impresión que usted preferiría como que la retaran en ese momento, o que le dijeran 'que tonta fuiste' (ríen) porque cuando yo le digo algo, usted me dice 'sí pero no, pero en realidad igual la embarré, no hay caso'; mmm parece que en este momento más le gustaría que yo me pusiera a criticarla mmm, como que incluso, si yo le digo algo así... como que usted también es inocente y que no es la responsable ehh es como tratarla de pobrecita, y más la complica ¿ah?" (T18S16:156).<br><br>*Therapist*: "Cierto, ¿y esa es una frustración para ti?" (T15S12:119).	*Therapist*: "You know? When I tell you these things I get the impression that you'd prefer to be reprimanded in that moment, or be told 'you've been such a fool' (laughs) because when I tell you something, you say 'yes, but no, but actually I screwed up anyway, there's no way around it'; um, it's as if even when I tell you something like... that you're also innocent and that you're not responsible um, it's sort of treating you like a poor little thing, and that makes you feel more uncomfortable, right?" (T18S16:156).<br><br>*Therapist*: "Right, and does that make you feel frustrated?" (T15S12:119).
**15**	(Resignifing=1) (Direction=1):	AND	SE	*Therapist*: "Porque ahí, yo creo que efectivamente, como un juego, tú dices algo sobre ella, como que ahí entonces la cosa se empieza a enredar, ya, vamos a dejar ese enredo para la otra semana" (T15S09:501).<br><br>*Therapist*: "Mire, yo la invitaría a pensar si eso que usted lanza con tanta fuerza... ¿no?, así como los hombres son unos 'básicos', digamos ¿no?, muy poco valiosos, de tenerlos antes en un pedestal, se me fueron abajo ehh me da a entender, como que tiene una muy mala opinión de ellos; yo estaba pensando si no será una forma de hablarme de usted al revés" (T10S2:519).	*Therapist*: "Because at that point, in fact, as part of a game, I think you say something about her, and then the issue starts getting complicated, okay, let's leave this mess for next week" (T15S09:501).<br><br>*Therapist*: "Look, I'd like to invite you to ask yourself whether those things you say with such force... right?, like 'men are such basic beings', right?, rather worthless... having been on a pedestal, they came tumbling down, um, with this you're telling me that you have a very bad opinion of them; I was thinking that maybe this is a way to talk to me about yourself in reverse" (T10S2:519).

REFERENCES

1 .Krause M, De la Parra G, Arístegui R, Dagnino P, Tomicic A, Valdés N, et al. The evolution of therapeutic change studied through generic change indicators. *Psychotherapy Research* **17** (2007) 673–689.

2 .Herrera Salinas P, Fernández González O, Krause Jacob M, Vilches Álvarez O, Valdés N, Dagnino P. Revisión teórica y metodológica de las dificultades en psicoterapia: propuesta de un modelo ordenador. *Terapia Psicológica* **27** (2009) 169–179.

3 .Quinlan JR. *C4.5: programs for machine learning* (Morgan Kaufmann) (1993).

4 .Kohavi R. Scaling up the accuracy of Naive–Bayes classifiers: a decision–tree hybrid. *Proceedings of the second international conference on knowledge discovery and data mining* (AAAI Press) (1996), 202–207.

5 .Quinlan JR. Learning with continuous classes. *Proceedings of the 5th Australian joint Conference on Artificial Intelligence* (Singapore) (1992), vol. 92, 343–348.
